# Early-Onset Aortic Dissection: Characterization of a New Pathogenic Splicing Variation in the *MYH11* Gene with Several In-Frame Abnormal Transcripts

**DOI:** 10.1155/2023/1410230

**Published:** 2023-08-14

**Authors:** Pauline Arnaud, Margaux Cadenet, Zakaria Mougin, Carine Le Goff, Sébastien Perbet, Mathilde Francois, Sophie Dupuis-Girod, Catherine Boileau, Nadine Hanna

**Affiliations:** ^1^Département de Génétique, AP-HP, Hôpital Bichat, F-75018 Paris, France; ^2^Université Paris Cité, Inserm, LVTS U1148, F-75018 Paris, France; ^3^Pôle de Médecine Péri-Opératoire, CHU Clermont-Ferrand, F-63000 Clermont-Ferrand, France; ^4^Service de Génétique, Hospices Civils de Lyon, Hôpital Femme-Mère-Enfant, F-69500 Bron, France

## Abstract

Rare pathogenic variants in the *MYH11* gene are responsible for thoracic aortic aneurysms and dissections. They are usually heterozygous missense variants or in-frame deletions of several amino acids without alteration of the reading frame and mainly affect the coiled-coil domain of the protein. Variants leading to a premature stop codon have been described in patients with another phenotype, megacystis-microcolon-intestinal hypoperistalsis syndrome, with an autosomal recessive inheritance. The physiopathological mechanisms arising from the different genetic alterations affecting the *MYH11* gene are still poorly understood. Consequently, variants of unknown significance are relatively frequent in this gene. We have identified a variant affecting the consensus donor splice site of exon 29 in the *MYH11* gene in a patient who suddenly died from an aortic type A dissection at the age of 23 years old. A transcript analysis on cultured fibroblasts has highlighted several abnormal transcripts including two in-frame transcripts. The first one is a deletion of the last 78 nucleotides of exon 29, corresponding to the use of a cryptic alternative donor splice site; the second one corresponds to an exon 29 skipping. Familial screening has revealed that this molecular event occurred *de novo* in the proband. Taken together, these experiments allowed us to classify this variant as pathogenic. This case underlines the challenging aspect of the discovery of variations in the *MYH11* gene for which the consequences on splicing should be systematically studied in detail.

## 1. Introduction

The thoracic aortic aneurysm corresponds to the dilation of the ascending part of the aorta, which can lead to a dissection or an aortic rupture (TAAD for thoracic aortic aneurysm and dissection). The etiologies are diverse, and a genetic origin is found in approximately 20% of the cases [1]. Hereditary TAADs are associated with significant mortality; for example, they contribute to approximately 20,000 deaths per year in the United States [2]. These pathologies are generally inherited in an autosomal dominant manner. They are characterized by great clinical and genetic heterogeneity with the involvement of more than thirty genes. They can be classified into syndromic and nonsyndromic forms, even if there is an overlap between these forms. The syndromic forms are more frequently linked to an alteration of a gene involved in the structure of the extracellular matrix or the TGF-*β* signaling pathway. The most representative are the Marfan syndrome linked to pathogenic variants in the *FBN1* gene (encoding fibrillin-1) and several disorders related to the Marfan syndrome (notably Loeys-Dietz syndrome) involving an alteration of the TGF-*β* signaling pathway. The nonsyndromic forms are more classically linked to a defect in the contractile apparatus of smooth muscle cells with, for example, pathogenic variants in the aortic smooth muscle *α*2-actin gene (*ACTA2*) or in the heavy chain of the myosin specific to smooth muscle (*MYH11*).

Pathogenic variants in the *MYH11* gene are rare and have been described as responsible for TAAD classically associated with patent ductus arteriosus [3–5]. This gene, located on the chromosome 16, encodes the heavy chain of the myosin specific to smooth muscle. Myosin is a contractile protein made up of two heavy chains and two pairs of light chains. The heavy chains twist together to form a helix. The myosin head is a globular structure that contains the actin-binding site and the catalytic site for ATP hydrolysis that allow the contraction of the smooth muscle cell [3–5]. The protein comprises 3 domains: the myosin head domain, the IQ (for isoleucine-glutamine) domain, and the coiled-coil domain. The *MYH11* heterozygous variants responsible for TAAD described in the literature are mainly located in the coiled-coil domain [6]. Classically, these variants are missense variants or in-frame deletions of several amino acids without alteration of the reading frame. This supports a probable dominant negative effect with the hypothesis that the presence of the variant protein affects the assembly of normal myosin molecules.

Interestingly, another phenotype, megacystis-microcolon-intestinal hypoperistalsis (MMIH) syndrome, is associated to *MYH11* gene with an autosomal recessive inheritance [7]. The homozygous and compound heterozygous variants associated with MMIH are mostly nonsense or frameshift variants that are predicted to result in premature termination codons [7–9]. In these recessive cases, the heterozygous carriers were not known to be affected with TAAD.

## 2. Material and Methods

### 2.1. Patient

The patient was a 23-year-old man who works as a nursing student in a medical intensive care unit. While he was at work, he suddenly died of a spontaneous aortic dissection of the ascending aorta (type A) without any particular cardiovascular risk factors. His parents were asymptomatic, without any particular family history of cardiovascular disease. A specific informed consent was obtained in agreement with the requirements of French bioethics laws.

### 2.2. DNA Amplification and Variant Detection

Genomic DNA was isolated from peripheral blood leukocytes with a DNA Blood 4K kit (Perkin Elmer®) on ChemagicSTAR (Hamilton®) according to the manufacturer's instructions. A custom capture array (NimbleGen, Roche®) designed to capture 28 genes involved in TAAD and Marfan-related disorders (*ACTA2* (NM_001613), *ADAMTSL4* (NM_019032), *BGN* (NM_001711), *COL3A1* (NM_000090), *EFEMP2* (NM_016938), *FBN1* (NM_000138), *FBN2* (NM_001999), *FLNA* (NM_001110556), *FOXE3* (NM_012186), *HCN4* (NM_005477), *LOX* (NM_002317), *LTBP2* (NM_000428), *LTBP3* (NM_001130144), *MAT2A* (NM_005911), *MFAP5* (NM_003480), *MYH11* (NM_001040113.2), *MYLK* (short aortic isoform corresponding to exons 18 to 34, NM_053025), *NOTCH1* (NM_017617), *PRKG1* (NM_006258), *SKI* (NM_003036), *SLC2A10* (NM_030777), *SMAD2* (NM_001003652), *SMAD3* (NM_005902), *SMAD4* (NM_005359), *TGFB2* (NM_001135599), *TGFB3* (NM_003239), *TGFBR1* (NM_004612), and *TGFBR2* (NM_003242)) was performed on NextSeq (Illumina®). The total size of the target was 132 kb. Variant calling is performed through CLC Genomics Workbench v10.1.1 (Qiagen® Bioinformatics). As described in a previous study [10], once a single nucleotide or a small insertion/deletion pathogenic variant is found in this way, it is systematically confirmed by bidirectional Sanger sequencing of the altered exon. When the pathogenic variant alters the regional restriction map, the presence of the variation is also checked by polymerase chain reaction (PCR)/digestion using the appropriate restriction enzyme (i.e., *BveI*). When possible, familial segregation of pathogenic variants is investigated. In the case of a *de novo* event, both maternity and paternity are confirmed by multiplex analysis of 16 highly polymorphic markers located on 14 different chromosomes (PowerPlex® 16 System (Promega®)). Description of sequence variants is performed according to Human Genome Variation Society nomenclature [11]. Effect on splicing was analyzed through different tools including Splice Site Finder-like (SSF-like [12]), MaxEntScan method [13], splicing prediction algorithm NNSPLICE [14], GeneSplicer [15], Splice AI [16], and SPiP [17]. The ClinVar database and the Genome Aggregation Database (gnomAD [18]) were searched for the existence of each molecular event.

### 2.3. Transcript Analysis

Patient skin fibroblasts were cultivated in DMEM (Thermo Scientific) supplemented with 4.5 g/L glucose, 15% fetal bovine serum, and antibiotics (50 U/mL of penicillin, streptomycin, and amphotericin B). Overnight incubation with emetine (Sigma-Aldrich®, 0.9 *μ*mol/L) was done in one flask to prevent nonsense-mediated mRNA decay (NMD). Total RNAs were extracted with the miRNeasy kit® (Qiagen S.A.) according to the manufacturer's instructions. After purification, RNAs were eluted in 20 *μ*L of RNase-free water, and RNA concentrations were estimated by measuring absorbance at 260 nm, using NanoDrop 2000/2000 c system (Thermo Scientific). Specific primers were designed within the exons 26 and 31 of the *MYH11* gene, with an expected normal size of amplification of 752 bp (M13-tailed primer sequences: Exon-26-F TGTAAAACGACGGCCAGT-AGAGCTCAAGATGCAGCTGG; Exon-31-R CAGGAAACAGCTATGACC-GCCGGGTTTCTTCTTGAAGC). 38 amplification cycles of RT-PCR were performed using Qiagen® One-Step RT-PCR Kit, according to the manufacturer's instructions. Amplification products were loaded for migration on a 10% acrylamide gel and on an agarose gel. The analysis was done after an image capture on ChemiDoc™ (Bio-Rad®). The bands corresponding to the different amplicons were cut out for standard bidirectional Sanger sequencing using the universal M13 forward and reverse primers.

## 3. Results

### 3.1. Molecular Results

**Figure 1:**
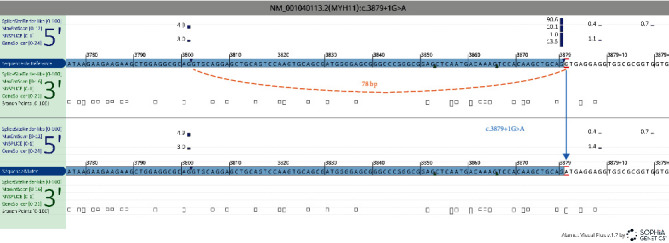
Schematic view of the predicted effect on splicing for the variation c.3879+1 in the *MYH11* gene *via* Alamut Visual Plus. Each line represents a splicing prediction algorithm: Splice Site Finder-like [12], MaxEntScan method [13], NNSPLICE [14], and GeneSplicer [15]. Donor splice sites are illustrated with blue squares. Note that a cryptic splice donor site is present 78 bp upstream the major donor splice site (illustrated in red).

Next-generation sequencing analysis retrieved in the patient's DNA a heterozygous variation in intron 29 of the *MYH11* gene: *MYH11* (NM_001040113.2): c.3879+1G>A (gDNA level: Chr16(GRCh37):g.15820704C>T). No other relevant variation was found in the other disease-causing genes. This variation was confirmed by bidirectional Sanger sequencing. The variation was identified for the first time in the laboratory (more than 4500 TAAD probands tested to date). It was also absent from the gnomAD population database. It was reported in a case of familial aortic aneurysm in the ClinVar database (ID 1467492). It was also described in a patient with aortic dissection, patent ductus arteriosus, and intracranial vessel stenosis [19]. By altering the consensus donor splice site, a missplicing is expected. This is confirmed by the use of the splicing predictors SSF-like, MaxEntScan, NNSPLICE, and GeneSplicer visualised *via* Alamut Visual Plus® (Figure 1). A cryptic splice donor site is present 78 bp upstream of the major donor splice site. Similar results were obtained using Splice AI and SPiP tools.

### 3.2. Transcript Analysis

**Figure 2:**
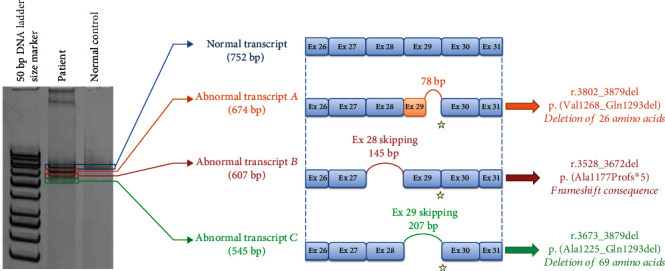
Results from the transcript analysis performed in the fibroblasts from negative control and patient (on the left) (amplification products on acrylamide gel). Schematic view of the different observed transcripts and the predicted consequence at protein level (on the right). The yellow star indicates the position of the variation observed on the patient DNA.

To characterize this splicing defect, a transcript study was carried out using a culture of fibroblasts from a skin biopsy of the patient. The acrylamide gel migration of the amplification products showed a band at 752 bp for the control, which represents the normal transcript. For the patient, gel migration showed two fairly intense bands at 674 bp and 752 bp as well as two bands of lower intensities at 607 bp and 545 bp (Figure 2). The amplification products were sequenced by bidirectional Sanger sequencing. Three alternative transcripts were highlighted in the patient. Two of the three transcripts lead to a defect with conservation of the reading frame at the protein level. These three transcripts affect the coiled-coil part of the protein. One corresponds to a deletion of the last 78 nucleotides of exon 29 leading to a loss of 26 amino acids, and it represents the majority transcript corresponding to the band at 674 bp on the gel (abnormal transcript A, r.3802_3879del, and p.(Val1268_Gln1293del)). The second abnormal transcript corresponds to the 607 bp band on the acrylamide gel (abnormal transcript B, r.3528_3672del, and p.(Ala1177Profs^∗^5)); it results from skipping of exon 28 leading in theory to the appearance of a premature termination codon. The third abnormal transcript corresponds to the skipping of the exon 29 leading to a loss of 69 amino acids and to the band at 545 bp on the gel (abnormal transcript C, r.3673_3879del, and p.(Ala1225_Gln1293del)).

### 3.3. Family Testing

**Figure 3:**
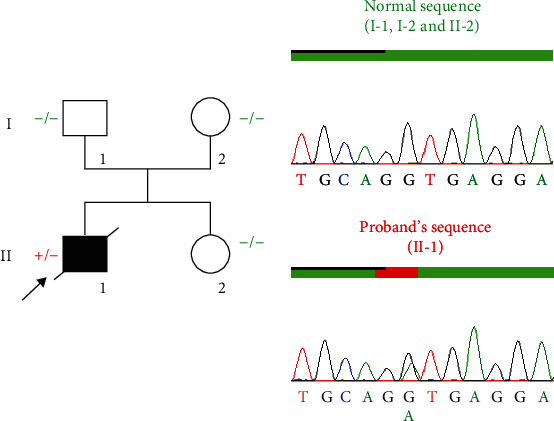
Pedigree of the family and results of targeted familial screening by bidirectional Sanger sequencing. An arrow indicates the proband. Only forward sequences are illustrated, and complementary results were obtained for the reverse sequences.

An analysis of the segregation of this variant with the phenotype in the family was necessary to determine whether it is involved in the pathology or not. No history of TAAD was reported in this family. The study of family segregation began with a complete clinical assessment of the patient's parents and sister. There is no dilation of any part of the aorta and no patent ductus arteriosus in both parents. The sister had a few skeletal signs such as wrist and thumb signs, flat feet, facial dysmorphism (dolichocephaly, enophthalmia, and malar hypoplasia), and a mitral valve prolapse. Genomic DNA isolated from peripheral blood lymphocytes from the parents and sister was analyzed by bidirectional Sanger sequencing. The results of the electropherograms clearly illustrate the absence of the variant in the parents and the sister of the patient (Figure 3).

**Figure 4:**
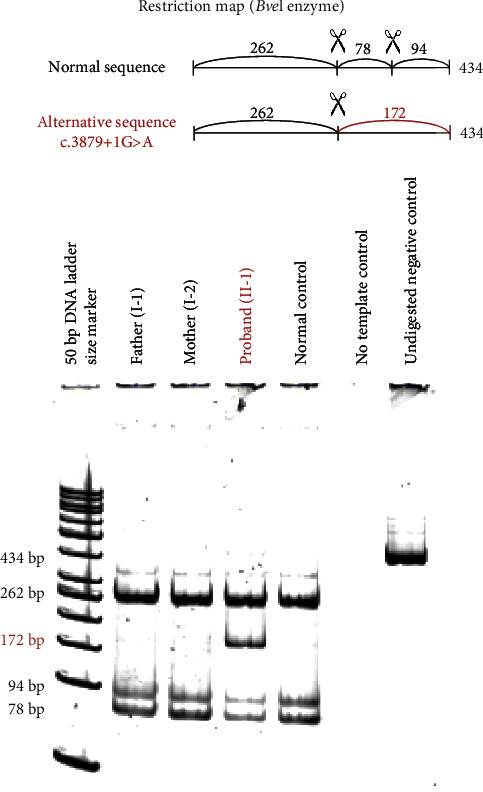
Enzymatic digestion of the DNA PCR amplification products in this family. The restriction map of the *BveI* enzyme is illustrated on the top of the figure. The enzymatic digestion was loaded onto a 10% acrylamide gel and captured with ChemiDoc™ system (Bio-Rad®). The alternative band of 172 bp corresponding to the loss of a restriction *BveI* site was only identified in the proband who carries the c.3879+1G>A variation.

In addition, the electropherograms and in particular the heights of the peaks were similar for both parents and the negative control, which suggests that there would be no detectable mosaicism in one of them. An enzymatic digestion of the PCR amplification products was then conducted in order to confirm the absence of mosaicism in the parents. A specific restriction enzyme with known restriction sites (*BveI*) was selected. The alternative band of 172 bp corresponding to the loss of a restriction *BveI* site was only identified in the proband who carries the c.3879+1G>A variation (Figure 4). No 172 bp was identified in the parents, even of low intensity, confirming the absence of detection of the variation as a mosaic. The paternity and maternity were verified using PowerPlex 16 analysis assuring that this variant occurred *de novo* in the patient.

## 4. Discussion and Conclusions

Few pathogenic variants in the *MYH11* gene are known to be responsible for TAAD [3–5] (9 different variants reported in UMD-MYH11 database, umd.be/MYH11/accessed on July 2023). Most of them have in-frame consequences leading to deletion of one or several amino acids at the protein level, suggesting a dominant negative effect. In-frame deletion of a string of amino acids may also have significant impact for protein folding, stability, or function. Nonsense or frameshift variants predicted to result in premature termination codons have been associated with megacystis-microcolon-intestinal hypoperistalsis (MMIH) syndrome, but with an autosomal recessive inheritance. In these families, heterozygous carriers are not known to be affected with TAAD [7–9], suggesting distinct physiopathology. Moreover, recurrent chromosome 16p13.1 duplications, including the entire *MYH11* gene, have been associated with an increased susceptibility of TAAD [20]. However, these copy number variations did not segregate with TAAD in the families and were relatively frequent in a population of controls (approximately 1 out of 1000 individuals). In summary, the physiopathological mechanisms arising from the different genetic alterations affecting *MYH11* gene are poorly understood. It is therefore essential to precisely characterize the variants identified in patients with TAAD in order to be able to provide an appropriate genetic counselling and attempt to refine the existing genotype-phenotype correlations.

We have identified a heterozygous variation in the *MYH11* gene that disrupts the donor splice site of exon 29 in a patient who died suddenly of an aortic type A dissection at the age of 23 years. At the protein level, this variation affected the coiled-coil domain of the protein, which has an important structural role [4, 6] and where most of the pathogenic variations were identified so far [5]. In the coiled-coil domain, the region encoded by exons 29 to 33 gathers most of the (likely) pathogenic variations identified so far. Thus, the predicted deletion of several amino acids of this critical region of the protein is likely to be pathogenic. The rarity of this variation and, in particular, its lack in the population database gnomAD supported its pathogenicity. Due to the suddenness of the dramatic event, the familial analysis was only possible with a delay.

Interestingly, this variation was already reported in the ClinVar database and in literature [19] in a case of aortic dissection associated with patent ductus arteriosus and intracranial vessel stenosis. It should be noted that in the case from the literature, the variation was inherited from the healthy mother. The sister's proband, who experienced an aortic aneurysm, was also carrying this variation. It was classified as likely pathogenic in ClinVar database, but no functional data was performed to investigate the consequences of this variation at mRNA level.

In the case of our study, a skin biopsy was performed in the proband. The RNA analysis on skin fibroblasts allowed the identification of at least two abnormal in-frame transcripts (abnormal transcripts A and C). As previously mentioned, the pathogenic variations in the *MYH11* gene previously described in TAAD are predicted to have in-frame consequences at the protein level, suggesting a dominant negative effect. Interestingly, the last abnormal transcript (abnormal transcript B, exon 28 skipping) leads in theory to a premature termination codon, which is not usually associated with TAAD. This experiment underlines the importance of the RNA analysis for the variations with an impact on splicing in this gene. Surprisingly, the presence of an abnormal transcript which skips the exon 28 illustrates the long-range consequences that can have a variation at the cDNA level since the variation was located in the intron 29 (distant from more 5700 bp at genomic level). The question of the type of sample on which to perform transcript analysis is central. According to the GTEx portal (https://www.gtexportal.org/home/, accessed in February 2023), the expression of the *MYH11* gene is high in the blood vessels, including aorta, but very low in the whole blood. In the experience of our laboratory, the RNA analysis of blood samples collected on PAXgene® is not efficient, especially for the *MYH11* gene, probably due to a very low expression in the blood. Some of these analyses may require nested PCR. A compromise is to use skin fibroblasts, in which the expression of the *MYH11* gene is intermediate. In our experience with other genes associated to TAAD, the expression in skin fibroblasts is a good mirror of what can happen at the aortic level [21]. Indeed, we manage to verify that the mRNA expression of the *FBN1* gene was similar for each subject in skin fibroblasts and adventitial aortic fibroblasts. The access to an aortic sample was impossible in the present case. In this study, we confirm that skin biopsy can be highly useful in the search for abnormal transcripts in the *MYH11* gene. However, the performed experiments on fibroblasts did not strictly demonstrate the accumulation and translation of the abnormal transcripts. A limitation of the study is the absence of protein study to highlight definitely the dominant negative effect.

Finally, the parental analysis allowed us to conclude that the molecular event occurred *de novo* in the proband. Taken together, all these arguments are in favor of the pathogenic nature of this variant according to American College of Medical Genetics and Genomics-Association for Molecular Pathology (ACMG-AMP) recommendations [22], using the following arguments: PS2, PS3, PM2, and PP3. The identification of this pathogenic variant in the proband confirms the diagnosis in him but also allows appropriate genetic counseling: the genetic risk is absent in his sister despite the mild skeletal features observed, and there is extremely low risk of recurrence for possible other future children of the couple. Interestingly, no patent ductus arteriosus was described in this proband, whereas it is a clinical feature which is regularly observed in the patients carrying pathogenic variations in the *MYH11* gene [3–5].

This case underlines a challenging aspect of the discovery of variations in the *MYH11* gene for which the consequences on splicing should be systematically studied in detail to conclude about the implication of the variants in the disease.

## Data Availability

The data used to support the findings of this study are available from the corresponding author upon request.
